# Overexpression of Transcription Factor Sp1 Leads to Gene Expression Perturbations and Cell Cycle Inhibition

**DOI:** 10.1371/journal.pone.0007035

**Published:** 2009-09-15

**Authors:** Emmanuelle Deniaud, Joël Baguet, Roxane Chalard, Bariza Blanquier, Lilia Brinza, Julien Meunier, Marie-Cécile Michallet, Aurélie Laugraud, Claudette Ah-Soon, Anne Wierinckx, Marc Castellazzi, Joël Lachuer, Christian Gautier, Jacqueline Marvel, Yann Leverrier

**Affiliations:** 1 Inserm, U851, Lyon, France; 2 Université Lyon1, IFR128, Lyon, France; 3 Université de Lyon, Lyon, France; 4 PRABI, Villeurbanne, France; 5 ProfileXpert, Bron, France; 6 Inserm, U758, Ecole Normale Supérieure de Lyon, Lyon, France; Lawrence Berkeley National Laboratory, United States of America

## Abstract

**Background:**

The ubiquitous transcription factor Sp1 regulates the expression of a vast number of genes involved in many cellular functions ranging from differentiation to proliferation and apoptosis. Sp1 expression levels show a dramatic increase during transformation and this could play a critical role for tumour development or maintenance. Although Sp1 deregulation might be beneficial for tumour cells, its overexpression induces apoptosis of untransformed cells. Here we further characterised the functional and transcriptional responses of untransformed cells following Sp1 overexpression.

**Methodology and Principal Findings:**

We made use of wild-type and DNA-binding-deficient Sp1 to demonstrate that the induction of apoptosis by Sp1 is dependent on its capacity to bind DNA. Genome-wide expression profiling identified genes involved in cancer, cell death and cell cycle as being enriched among differentially expressed genes following Sp1 overexpression. *In silico* search to determine the presence of Sp1 binding sites in the promoter region of modulated genes was conducted. Genes that contained Sp1 binding sites in their promoters were enriched among down-regulated genes. The endogenous *sp1* gene is one of the most down-regulated suggesting a negative feedback loop induced by overexpressed Sp1. In contrast, genes containing Sp1 binding sites in their promoters were not enriched among up-regulated genes. These results suggest that the transcriptional response involves both direct Sp1-driven transcription and indirect mechanisms. Finally, we show that Sp1 overexpression led to a modified expression of G1/S transition regulatory genes such as the down-regulation of *cyclin D2* and the up-regulation of *cyclin G2* and *cdkn2c/p18* expression. The biological significance of these modifications was confirmed by showing that the cells accumulated in the G1 phase of the cell cycle before the onset of apoptosis.

**Conclusion:**

This study shows that the binding to DNA of overexpressed Sp1 induces an inhibition of cell cycle progression that precedes apoptosis and a transcriptional response targeting genes containing Sp1 binding sites in their promoter or not suggesting both direct Sp1-driven transcription and indirect mechanisms.

## Introduction

Transcription factor Sp1 was the first identified member of the Sp/XKLF (Specificity protein/Krüppel-like factor) family. Sp1 protein comprises several domains of which the DNA binding domain is the most conserved among Sp family. The DNA binding domain of Sp1 consists of three contiguous Cys_2_His_2_ Zinc (Zn) fingers and mutational analysis has revealed that Zn fingers 2 and 3 are essential for Sp1 DNA binding activity [1]. Sp1 binds GC-rich elements [2] that are common regulatory elements in promoters of numerous genes. Sp1 binds individual Sp1 binding sites as a multimer and is capable of synergic activation on promoters containing multiple binding sites [3]. Sp1 regulates transcription by dynamically recruiting and forming complexes with many factors associated with transcription [4]. Although Sp1 has been described as a transcriptional activator it can also act as a repressor. Activation or repression of transcription by Sp1 depends on the promoter it binds to and on the co-regulators it interacts with [5].

An unbiased mapping of *in vivo* occupied Sp1 binding sites by combining chromatin immunoprecipitation and oligonucleotides arrays has led to the estimation that the human genome contains at least 12,000 Sp1 binding sites [6]. Therefore it is not surprising that Sp1 has been implicated in the expression of numerous genes involved in many aspects of cellular life such as metabolism, cell growth, differentiation, angiogenesis and apoptosis regulation. Although Sp1 is widely expressed and binds the promoters of a large number of genes, it is involved in tissue specific gene expression, its activity being finely modulated by a variety of stimuli through multiple post-translational modifications [7].

Sp1 expression levels are also regulated, changes in its expression levels being observed during murine development and during transformation. Indeed, differences in the levels of Sp1 of up to 100 times were observed during the development and the differentiation of mouse organs [8]. Importantly, Sp1 expression is increased in a number of tumour cells and this could be a critical factor for tumour development or maintenance. Indeed, Sp1 levels and/or activities are increased in gastric cancer, breast carcinoma and pancreatic carcinoma compared with normal tissues [1], [9], [10]. This elevated Sp1 expression is inversely correlated with the survival of patients with gastric cancer [9]. In primary pancreatic adenocarcinoma Sp1 overexpression identifies advanced stage tumours and predicts a poor clinical outcome [11]. Moreover, Sp1 levels slowly increase during mice skin tumour progression [12] and Sp1 accumulates in N-methyl-N-nitrosourea-induced mammary tumour cells compared to normal mammary cells [13]. Sp1 levels also increase during the process of transformation in a fibrosarcoma transformation model and reducing Sp1 expression in those human transformed fibroblasts inhibits their tumorogenicity [14]. Moreover reduction of Sp1 expression in pancreatic cancer cells inhibit their growth and metastasis in mouse models [15]. Sp1 could contribute to transformation via the regulation of expression of genes regulating cell growth (*c-jun*, *Raf*, cyclins, cdk inhibitors, *E2F1*, *TGF-* ***β***, *IEX-1* and *TCL1*), apoptosis (*Bcl-2*) or angiogenesis (*VEGF, FGF*) [16], [17]. Altogether, these findings show that Sp1 is overexpressed or overactivated in a number of cancers and that its activity plays a role in late stage of carcinogenesis.

Although Sp1 deregulation might be beneficial for tumour cells, we and others have previously shown that deregulation of Sp1 expression on its own induces apoptosis of several untransformed cell lines [18], [19]. The aim of the study was to further characterise the functional and transcriptional responses of untransformed cells following Sp1 overexpression. We used wild-type and DNA-binding-deficient Sp1 to demonstrate that Sp1-induction of apoptosis in untransformed Baf-3 cells requires its binding to DNA. Genome-wide expression profiling showed that Sp1 overexpression induces a transcriptional response that is enriched for genes regulating cell death and cell cycle. Sp1 overexpression leads to the down-regulation of *cyclin D2* expression and the up-regulation of *cyclin G2* and *cdkn2c* expression. Moreover, we show that this deregulation of cell cycle regulating genes is associated with the accumulation of cells in the G1 phase of the cell cycle. Finally, microarray data combined with promoter analysis revealed that only a fraction of the promoters of deregulated genes are enriched in Sp1-binding sites. This suggests that the transcriptional response induced by overexpressed Sp1 involves direct Sp1-driven transcription but also indirect mechanisms.

## Materials and Methods

### Cell culture

**Figure 1:**
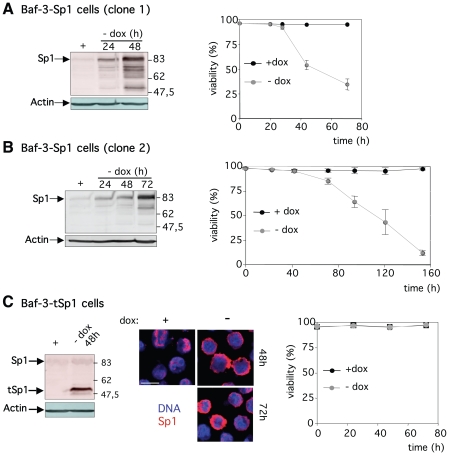
Deregulation of the expression of wild-type but not truncated cytoplasmic Sp1 induces apoptosis. (A) Baf-3-Sp1 clone 1 and (B) Baf-3-Sp1 clone 2 were grown in presence of doxycycline (+ dox) or in absence of doxycycline for the indicated time (- dox). Analysis of Sp1 and actin protein levels by Western Blot (*left panel*). Viability measured by flow cytometry after staining with propidium iodide (*right panel*). (C) Inducible clone Baf-3-tSp1 was grown with (+ dox) or without (-dox) doxycycline for the indicated time. Analysis of Sp1 and actin protein levels by Western Blot (*left panel*). tSp1: truncated Sp1. Cells were costained for DNA (blue) and Sp1 (red) and analysed by confocal microscopy (*middle panel*). Scale bar 10 µm. Viability measured by flow cytometry after staining with propidium iodide (*right panel*). Results show the mean ± sd of 3 independent experiments.

The murine IL-3-dependent Baf-3 cell line was maintained in DMEM (Gibco) containing 6% FBS (Pan Biotech GmbH) and 5% IL-3 as described [20]. Drosophila Schneider's SL2 cells were cultured in Drosophila medium (Invitrogen) containing 10% FBS at 28°C [21]. Baf-3 clones expressing inducible Sp1 and GFP were obtained as described previously [18]. To repress ectopic Sp1 expression, cells were grown in presence of doxycyline (30 ng/ml, Sigma). To induce Sp1 and GFP expression, cells were washed 3 times and cultured without doxycyline. Among the inducible clones generated, one expressed a truncated form of Sp1 (tSp1, Figure 1C) due to the integration into the genome of a retrovirus coding for a truncated Sp1 (data not shown). tSp1 is composed of the first 418 amino acids and is devoid of DNA binding domain and nuclear localisation sequences (Figure S1).

### Retroviral constructions, transfections and transductions

The retroviruses coding for full-length human Sp1 protein followed by an internal ribosomal entry site and a truncated cell surface marker (CD2) to identify transduced cells were described previously [18]. Sp1 carrying mutations of the second and third Zn Fingers (Sp1^Zn2,3^) (Figure S1) was obtained by PCR-mediated site-directed mutagenesis using pCMX-Gal4NSp1ZFM2 vector (a kind gift of Dr Hur MW, Seoul, Korea) encoding the DNA binding domain (amino acids 622–720) mutated for the second Zn Finger. Mutation of the third Zn finger was obtained using the following oligonucleotides 5′-CCTCATGAAGCGCTTAGGACTCTCAGGGCTGGCAAATTTCTTCTCACCTGTGTGTGTACGTTTGTGCCTCTGTAGCTCATCGGTGAGAAGAAATTTGCCAGCCCTGAGTGCCTAAG-3′, BamHI site: 5′-CCGCGGATCCTGGCAAAAAGAAACAGC-3′. The BamHI/Ecor47III cDNA PCR fragments were subcloned into pBSK-Sp1 to give pBSK-Sp1Zn^2,3^. The EcoRI-XhoI fragment was sequenced before subcloning into pMX-IRES-CD2. Sp1, Sp1^Zn2,3^ or EGFP cDNA were cloned under the Drosophila actin AC5 promoter into pAc5.1/V5-HisA vector (Invitrogen). The pGL2 derivative carries the tata-Sp1 promoter [21]. Transfections, transductions, magnetic selection of CD2-expressing cells and western blot analysis were performed as described [18].

### Luciferase assay

SL2 cells were transfected with 5 µg of tata-Sp1 reporter plasmid and 0.5 µg of Drosophila expression vectors pPac, pPacSp1 or pPacSp1^Zn2,3^ and 0.5 µg of pPac-EGFP to quantify transfection efficiency. 48 hrs after transfection cells were lysed in 100 µl of Reporter Lysis Buffer (Promega) for 10 min. Samples were frozen at −80°C for at least 1 h. Luciferase assay was performed with Luciferase Assay System (Promega). Luciferase activity was normalized based on protein concentration (Bradford method) and on transfection efficiency determined as percentage of EGFP positive cells counted on a FACScalibur (Becton-Dickinson) and analysed using the CellQuest software.

### Electrophoretic mobility shift assay

Proteins Sp1 or Sp1^Zn2,3^ were synthesised *in vitro* using a reticulocyte coupled transcription/translation system (Promega). The oligonucleotide probe carrying the Sp1 consensus sequence (upper strand sequence: 5′-ATTCGATCGGGGCGGGGCGAGC-3′) was ^32^P end-labelled with T4 DNA polynucleotide kinase (Promega). The binding reaction was performed in a 15 µl volume of binding buffer containing 2.5 µl of *in vitro* protein, 1.5 µg poly dI-dC and oligonucleotide probe for 30 min at room temperature and run on a gel.

### Cell viability and cell cycle analysis

Viability was measured using Fluorescein-5-isothiocyanate (FITC)-Annexin V staining (BD Biosciences Pharmingen) or Propidium Iodide staining (2 µg/ml, Sigma) by flow cytometry. Cell cycle was analysed using the Flow Cytometry Kit (BD Biosciences Pharmingen). Cells (2.10^5^ cells/ml) were grown for 16 hrs and then labelled with BrdU (32 µM, 20 min). Cells were fixed and permeabilised with Cytofix/Cytoperm and treated with DNase (1 hr, 37°C). Cells were stained with Allophycocyanin-conjugated anti-BrdU and 7-amino-actinomycin D (7-AAD). When required, transduced cells were stained with Phycoerythrin (PE)-coupled anti-CD2 antibody (30 min, 4 µg/ml, BD Biosciences Pharmingen). BrdU incorporation (FL4-H), 7-AAD (FL3-H) and CD2 expression (FL2-H) were recorded using a FACScalibur.

### Immunofluorescence microscopy

Cells seeded on 12-mm glass coverslips coated with 0.01% Poly-L-lysine (Sigma) were fixed with 4% paraformaldehyde (BDH) in PBS for 5 min then permeabilised with 0.25% Triton X-100 (BDH) in PBS for 5 min. Sp1 was detected using anti-Sp1 polyclonal rabbit antibody (1∶100, 120 min) followed by biotin-conjugated goat anti-rabbit antibody (5 µg/ml, Jackson ImmunoResearch, 30 min) and either FITC-conjugated streptavidin (1 µg/ml, BD Biosciences Pharmingen, 30 min) or Tetramethyl-Rhodamine-Iso-Thio-Cyanate (TRITC)-conjugated streptavidin (3 µg/ml, Jackson ImmunoResearch, 30 min). Actin was labelled with rhodamine-conjugated phalloidin (0.2 µg/ml, Sigma, 30 min).

Detergent Triton X-100 was used to remove soluble proteins while leaving more tightly bound protein [22]. Thirty hrs after transduction, 10^6^ cells were stained for 45 min with 0.5 µg biotinylated anti-CD2 antibody and with 0.75 µg TRITC-conjugated streptavidin for 20 min (Jackson ImmunoResearch). Cells were then washed in PBS containing 0.5 mM MgCl_2_ and 0.5 mM CaCl_2_, incubated for 5 min in CSK buffer (10 mM Pipes-KOH, pH 7.0, 100 mM NaCl, 300 mM sucrose, 3 mM MgCl_2_) and incubated for 3 min in CSK buffer containing 0.02% Triton X-100. A 10 times excess of CSK buffer was added and cells were loaded on Poly-L-lysine-coated coverslips and fixed immediately with 4% paraformaldehyde. Specimens were stained with 1∶100 anti-Sp1 antibody for 2 hrs followed by Alexa488 conjugated anti-rabbit antibody (Molecular Probes). Actin staining was performed in parallel to confirm the efficiency of triton removal of soluble proteins (not shown). Slides were mounted with ProLong anti-fade (Molecular Probes) containing DRAQ5 (1∶500, Alexis corporation) to counterstain DNA. Images of cells (single sections) were obtained using a Zeiss LSM 510 confocal laser-scanning microscope. Sp1 staining in CD2-positive cells was quantified using Image J software. The ratio of Sp1 signal in transduced CD2 positive cells versus non transduced neighbouring cells was calculated.

### Quantification by Real Time PCR

RNA extraction and reverse transcription were performed as previously described [18]. Real Time PCR analysis was carried out using Platinum SYBR Green qPCR SuperMix UDG kit (Invitrogen) on an ABI Prism 7700 (Perkin Elmer) [23]. Relative level of the target sequence against the HPRT reference sequence was calculated using the ΔΔCt method with calculated real efficiencies. The sequences of primers used are:

Neo:Forward-ACCAAGGAAGGGGTTGCTA,Reverse-GTTTTGAATAGTCACCTGCATCTC;

Cxcl4:F-GCGTCGCTGCGGTGTTTCG,R-ATCCCAGAGGAGATGGTCTTCACAC;

Slc39a8:F-TTTCGTGGGACTCGCTATTGGG,R-ATCCACCAAACACAGCAACTGC;

Jun:F-AAGAACTGCATGGACCTAACATTCG,R-GTTAAGGAGCACTACAGAAGCAATCTAC;

Gsn:F-AGGTCAAAGGACGCCGTGTAG,R-GCCAGAGCCACACCACTGATAG;

Ccnd2:F- TTCATTGAGCACATCCTTCGC,R-AAGTCGGTAGCGCACAGAGC;

Ccng2:F-GCCGAGTTGTCTTCTCCAAAGC,R-AAGCAAGAGAATTTCCAGCAGTTCC;

Cdkn2c:F-GTCCTTCTGTCAGCCTCCGATG,R-TCGGCCATTCTTTAGGGTCCTG;

Human Sp1:F-GCCTCCAGACCATTAACCTCAGT,R-GCTCCATGATCACCTGGGGCAT;

Mouse Sp1: F-TCATGGATCTGGTGGTGATGGG,R-GCTCTTCCCTCACTGTCTTTGC;

Hprt1:F-TCATTATGCCGAGGATTTGGA,R-CAGAGGGCCACAATGTGATG.

### Microarray analysis of gene expression

Inducible Baf-3-Sp1 clone 1 was grown in presence or absence of doxycycline for 28 hrs. Three independent experiments were performed. RNA extraction, reverse transcription and microarray hybridization on Affymetrix mouse Genechip Mouse genome 430 2.0 (Affymetrix) were carried out by ProfileXpert (Bron, France). Absolute expression transcripts levels were normalized for each chip by globally scaling all probesets to a target signal intensity of 500. Normalized data were analysed using GeneSpring GX 7.3 expression analysis software (Agilent Technology Inc). Differential probesets expression following Sp1 overexpression relative to control was determined using GeneSpring GX 7.3 statistical analysis module ANOVA (1 way test, assuming variance not equal, FDR 6% with multiple testing correction Benjamini and Hochberg). 1610 probesets corresponding to 1294 genes with unique gene symbol identifiers were differentially expressed with a fold change of expression of 1.3. To identify among deregulated genes the functional categories linked with Sp1 overexpression, differentially expressed probesets were imported into Ingenuity Pathway Analysis (Ingenuity Systems Inc, Mountain View, CA). The microarray data have been deposited and described in ArrayExpress under accession number E-MEXP-1702 (http://www.ebi.ac.uk/microarray-as/ae) in accordance with MIAME guidelines.

### Computational analysis of promoters regions

Promoter sequences (2000 nucleotides upstream of the transcriptional start site) were retrieved using Biomart service of the Ensembl project. Elkon et al. have shown that 80% of Transcription Factor-binding sites are located within 1200 bases upstream of the transcription start site (TSS) [24]. Since there is no clear consensus for the length of a promoter, we have performed our analysis studying the 2000 bases upstream of the TSS. Sp1 binding motifs GGGGCGGGG (V$SP1_Q6 as defined by TRANFAC 7.0) and GG[G/T]G[C/T]GGG [6] and STAT6 binding motif (TTCN_4_GAA) [25] were searched in promoters using PATTERNn service (http://bioinfo.hku.hk/services/analyseq/cgi-bin/patternn_in.pl). We have also used the DiRE server (http://dire.dcode.org) to identify transcription factor binding sites within conserved Regulatory Elements (RE) of deregulated genes. A control list of 1700 probesets was generated randomly that fulfils two criteria: no change in their expression levels following Sp1 overexpression and an overall distribution of expression of the control list similar to the overall distribution of expression of the differentially expressed list (Figure S2). Two sided binomial tests were conducted in R software to determine whether there were significant differences in the percentage of promoters containing transcription factor binding site between the different gene lists.

## Results

### Induction of apoptosis by overexpressed Sp1 requires its binding to DNA

The overexpression of transcription factor Sp1 was achieved using an inducible Sp1 expression system in untransformed murine haematopoietic Baf-3 cell line [18]. In Baf-3-Sp1 clone 1 the expression of ectopic full-length wild-type Sp1 was rapidly induced following doxycycline removal (Figure 1A). The first cells entering apoptosis were detected 30 hrs after doxycycline removal. Similar results were obtained when studying a second inducible clone (Baf-3-Sp1 clone 2) in which induction of Sp1 expression and apoptosis induction following doxycycline removal are slower (Figure 1B). In contrast a truncated Sp1 (tSp1, Figure S1), that accumulates in the cytoplasm does not affect cell viability (Figure 1C).

**Figure 2:**
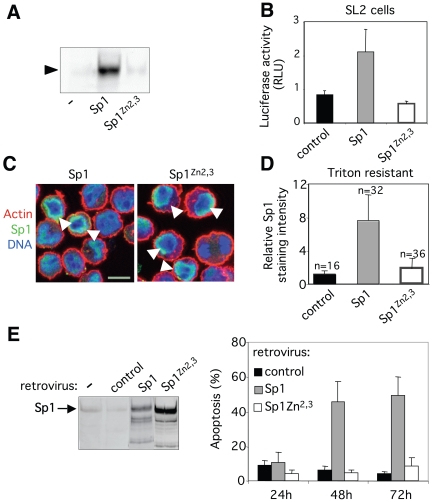
DNA binding deficient Sp1^Zn2,3^ does not induce apoptosis. (A) EMSA without (-) or with *in vitro* produced wild-type human Sp1 (Sp1) and mutant Sp1 carrying point mutations on Zn fingers 2 and 3 (Sp1^Zn2,3^) using ^32^P-labeled oligonucleotide carrying a Sp1 consensus sequence as a probe. Arrowhead indicates the DNA-protein complex. (B) Drosophila SL2 cells were transiently co-transfected with luciferase reporter plasmid pGL2 driven by the *tata-Sp1* promoter and plasmids pAC encoding wild-type Sp1 or mutated Sp1Zn2,3. Empty vector was used as control and pPac-EGFP to normalize the efficiency of transfection. Luciferase activity was assayed 48 hrs after transfection. Results are expressed as relative light units. Results are the mean ± sd of triplicate samples and are representative of at least 2 independent experiments. (C) Baf-3 cells were transduced with retroviruses encoding wild-type Sp1 or Sp1^Zn2,3^ and analysed 30 hrs later. Cells were costained for actin (red), Sp1 (green) and DNA (blue) and analysed by confocal microscopy. Arrowheads indicate cells overexpressing Sp1 or Sp1^Zn2,3^. Scale bar, 10 µm. (D) Quantification of DNA-bound Sp1 (Triton resistant). Twenty-four hrs post-infection with control, Sp1 or Sp1^Zn2,3^ carrying retroviruses, cells were stained for CD2, submitted to extraction with triton to remove soluble proteins and fixed. Cells were then stained for Sp1. The intensity of Sp1 staining was quantified in transduced CD2-positive cells (n) as well as in non-transduced neighbouring cells. The ratio of Sp1 signals in transduced versus non-transduced cells is presented. (E) Western blot analysis of Sp1 protein levels in parental Baf-3 cells and in cells transduced with either control, Sp1 or Sp1^Zn2,3^ retroviruses purified by magnetic selection with anti-CD2 antibody 30 hrs post-infection (*left panel*). The percentage of apoptotic cells (Annexin V positive) was measured at various time points among transduced cells (CD2 positive) by flow cytometry (*right panel*). Results show the mean ± sd of 3 independent experiments.

We then assessed whether Sp1 requires nuclear localisation and/or DNA binding to induce apoptosis. Therefore, we have constructed a retrovirus coding for Sp1 carrying point mutations on the Zn fingers two and three (Sp1^Zn2,3^) that are essential for DNA binding [1] (Figure S1). Sp1^Zn2,3^ does not bind Sp1-binding consensus sequence *in vitro* as revealed by EMSA analysis (Figure 2A). The lack of transactivation potential of Sp1^Zn2,3^ mutant was first verified in Drosophila Schneider SL2 cells since these cells are devoid of Sp-like activities. In SL2 cell line, wild-type Sp1 transactivates a promoter carrying Sp1 binding sites confirming that this form is transcriptionally active (Figure 2B). As expected Sp1^Zn2,3^ does not transactivate the same promoter. As proteins of the Sp/KLF family have nuclear localisation sequences adjacent to or within the Zn-finger motifs we next monitored the localisation of transduced Sp1^Zn2,3^ protein. Both Sp1 and Sp1^Zn2,3^ are localised in the nucleus of Baf-3 cells (Figure 2C). The binding of Sp1^Zn2,3^ to chromatin was assessed using triton extraction on live cells [22]. This approach which allows the removal of soluble proteins but not DNA-associated proteins was followed by immunostaining of Sp1 to quantify DNA-bound Sp1. Endogenous and wild-type ectopic Sp1 are triton-resistant confirming their association with DNA whereas Sp1^Zn2,3^ is triton-soluble suggesting a lack of a strong association with DNA (Figure 2D). When overexpressed in Baf-3 cells, wild-type Sp1 induces apoptosis whereas Sp1^Zn2,3^ mutant does not (Figure 2E). Mutations of the Zn finger 2 (Sp1^Zn2^), Zn finger 3 (Sp1^Zn3^) or the three Zn fingers (Sp1^Zn1,2,3^) also abrogate Sp1 ability to induce apoptosis (Figure S3). Altogether, these results show that induction of apoptosis by Sp1 requires its ability to bind to DNA through its Zn fingers and suggest that a transcriptional process is involved.

### Transcriptional response associated with overexpression of Sp1

**Figure 3:**
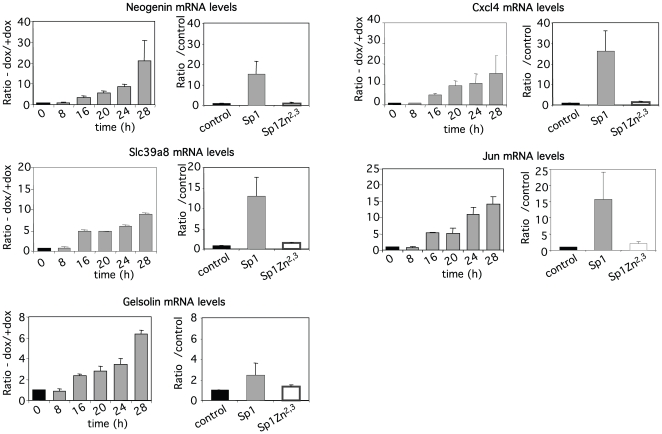
Changes in expression profile is dependent on Sp1 binding to DNA. Kinetic of expression levels of five differentially expressed genes (Neogenin, Slc39a8, Gelsolin, Cxcl4, Jun) following doxycycline removal in Baf-3-Sp1 clone 1 (*left panel*). At the indicated times, cells were harvested and mRNA levels were measured by real-time PCR and normalized to HPRT mRNA levels. Results show the ratio of the mRNA levels measured in the absence of doxycycline relative to mRNA levels measured in the presence of doxycycline at each time point. Results show the mean ± sd of at least 2 independent experiments. Expression levels in Baf-3 cells transduced with either control, Sp1 or Sp1^Zn2,3^ retroviruses purified by magnetic selection with anti-CD2 antibody 30 hrs post-infection (*right panel*). Results show the mean ± sd of at least 2 independent experiments.

To have a better insight into the molecular perturbations induced by Sp1 overexpression, we performed a genome-wide expression profiling to identify set of genes that are affected by overexpression of Sp1. Expression profiling was conducted comparing Baf-3-Sp1 clone 1 grown in the presence of doxycycline for 28 hrs as control and the same clone grown 28 hrs without doxycycline. This time corresponds to a point where cells already expressed high levels of Sp1 protein without any detectable cell death (Figure 1A). Three independent experiments were performed and analysed using the Affymetrix genechip Mouse genome 430 2.0 arrays (data deposited in ArrayExpress http://www.ebi.ac.uk/microarray-as/ae, accession number E-MEXP-1702). Sp1 overexpression was associated with a substantial modification of transcription profiles (Figure S4). Differential probesets expression following Sp1 overexpression relative to control was determined using statistical analysis in GeneSpring: 1294 genes with unique gene symbol identifiers were found to be differentially expressed with a fold change of expression of 1.3. Among them, the expression of 766 genes was increased whereas the expression of 528 genes was decreased. With a two-fold change of expression, 172 genes with unique gene symbol identifiers were found to be differentially expressed (162 up-regulated genes, 10 down-regulated genes). Table 1 shows the most up-regulated and down-regulated genes. Sp1 overexpression has an effect on the expression of many genes involved in metabolism, ubiquitination and transcription but also many genes with unknown functions. The kinetic of expression of five of the most up-regulated genes (*Neo1*, *Cxcl4*, *Gsn*, *Slc39a8,* and *Jun*) was precisely monitored using quantitative real-time PCR (Figure 3, left panels). Their up-regulation starts to be detected 16 hrs following doxycycline removal and is also observed in Baf-3 cells transduced with wild-type Sp1 but not Sp1^Zn2,3^ indicating that the binding of Sp1 to DNA is necessary for their induction (Figure 3, right panels).

**Table 1:** Genes affected by Sp1 overexpression.

Functional category	Symbol	Full name	Fold Change	UniGene	Ensembl	Affymetrix ID
Metabolism						
	Upp1	uridine phosphorylase 1	5,0	Mm.4610	ENSMUSG00000020407	1448562_at
	St8sia4	ST8 alpha-N-acetyl-neuraminide alpha-2,8-sialyltransferase 4	3,6	Mm.306228	ENSMUSG00000040710	1430391_a_at
	Galnt3	UDP-N-acetyl-alpha-D-galactosamine:polypeptide N-acetylgalactosaminyltransferase 3	3,4	Mm.38441	ENSMUSG00000026994	1417588_at
	Slc39a8	solute carrier family 39 (metal ion transporter), member 8	3,3	Mm.30239	ENSMUSG00000053897	1416832_at
	Tgm2	transglutaminase 2	3,1	Mm.330731	ENSMUSG00000037820	1433428_x_at
	Mtap	methylthioadenosine phosphorylase	−2,1	Mm.28500	ENSMUSG00000062937	1424426_at
Ubiquitin						
	Arih1	ariadne ubiquitin-conjugating enzyme E2 binding protein homolog 1	5,5	Mm.305925		1442730_at
	Fbxw2	F-box and WD-40 domain protein 2	3,2			1454002_at
	Pja2	praja 2	−2,0	Mm.41711	ENSMUSG00000024083	1427148_at
Transcription						
	Jun	Jun	3,8	Mm.275071		1417409_at
	Irf7	interferon regulatory factor 7	3,5	Mm.3233	ENSMUSG00000025498	1417244_a_at
	Bcl9	B-cell CLL/lymphoma 9	−2,0	Mm.87600	ENSMUSG00000038256	1451574_at
	Sp1	trans-acting transcription factor 1	−2,3	Mm.4618		1454852_at
	Mecp2	methyl CpG binding protein 2	−2,5	Mm.131408	ENSMUSG00000031393	1438930_s_at
Adhesion/Migration						
	Gsn	Gelsolin	6,2	Mm.21109	ENSMUSG00000026879	1437171_x_at
	Arl6ip1	ADP-ribosylation factor-like 6 interacting protein 1	5,3	Mm.29924	ENSMUSG00000030654	1423819_s_at
	Prickle1	prickle like 1	3,4	Mm.150314		1452249_at
Receptors/transmembrane						
	Neo1	neogenin	10,1	Mm.42249	ENSMUSG00000032340	1447693_s_at
	Tmem51	transmembrane protein 51	6,5	Mm.27587	ENSMUSG00000040616	1424383_at
	Tmem71	transmembrane protein 71	6	Mm.132299	ENSMUSG00000036944	1436212_at
	Tnfrsf22	tumor necrosis factor receptor superfamily, member 22	3,9	Mm.261384	ENSMUSG00000010751	
	Tmem173	transmembrane protein 173	3,7	Mm.45995	ENSMUSG00000024349	1447621_s_at
	Tnfrsf9	tumor necrosis factor receptor superfamily, member 9	3,4	Mm.244187	ENSMUSG00000028965	1428034_a_at
	Clec2d	C-type lectin domain family 2, member d	−2,3	Mm.197536	ENSMUSG00000030157	1419477_at
Apoptosis						
	Bmf	Bcl2 modifying factor	4,0	Mm.210125	ENSMUSG00000040093	1454880_s_at
Cell cycle						
	Lats2	large tumor suppressor 2	3,7	Mm.347899	ENSMUSG00000021959	1439441_x_at
Exocytosis/Trafficking						
	Stxbp6	syntaxin binding protein 6 (amisyn)	7,4	Mm.285400		1435396_at
	Cd74	class II antigen-associated, Ii	3,9	Mm.276499	ENSMUSG00000024610	1425519_a_at
	Tcte1l	dynein light chain Tctex-type 3	−2,0	Mm.29150	ENSMUSG00000031176	1449929_at
Chemokine						
	Pf4 (Cxcl4)	chemokine (C-X-C motif) ligand 4	8,4	Mm.332490	ENSMUSG00000029373	1448995_at
Inflammation						
	Gbp1	guanylate nucleotide binding protein 1	7,8	Mm.250	ENSMUSG00000028269	1420549_at
	Gbp2	guanylate nucleotide binding protein 2	5,1	Mm.24038	ENSMUSG00000028270	1418240_at
	Serpinf1	serine (or cysteine) peptidase inhibitor, clade F, member 1	4,3	Mm.2044	ENSMUSG00000000753	1416168_at
Signal transduction						
	Pik3r3	phosphatidylinositol 3 kinase, regulatory subunit, polypeptide 3 (p55)	5,9	Mm.253819	ENSMUSG00000028698	1456482_at
	Eps8	epidermal growth factor receptor pathway substrate 8	3,4	Mm.235346	ENSMUSG00000015766	1422824_s_at
	Ppp3cb	protein phosphatase 3, catalytic subunit, beta isoform	−2,1	Mm.274432	ENSMUSG00000021816	1433835_at
Misclaneous						
	Hist2h2aa2	histone cluster 2, H2aa2	11,0			1438815_at
	E230032D23Rik	E230032D23Rik	6,5	Mm.387210		1444420_at
	4933411B09Rik	4933411B09Rik	4,7			1430863_at
			4,2			1443777_at
	RNAse k	ribonuclease, RNase K	4,0	Mm.29852	ENSMUSG00000040904	1442441_at
	Ccdc19	coiled-coil domain containing 19	3,6	Mm.78373	ENSMUSG00000026546	1429930_at
	3110040M04Rik	3110040M04Rik	3,5	Mm.359303		1429700_at
			3,5	Mm.75540		1447844_at
	Calml4	calmodulin-like 4	3,4	Mm.28623	ENSMUSG00000032246	1424713_at
	4930513N10Rik	4930513N10Rik	3,2	Mm.386996		1457415_a_at
	Scoc	short coiled-coil protein	−2,0	Mm.379079	ENSMUSG00000063253	1416267_at
	Morc3	microrchidia 3	−2,1	Mm.287329	ENSMUSG00000039456	1452224_at
	Hba-a1	hemoglobin alpha, adult chain 1	−2,3	Mm.196110	ENSMUSG00000069917	1452757_s_at

Genes identified by microarray analysis (Affymetrix GeneChip Microarray 430.2) as most up-regulated or down-regulated following ectopic Sp1 expression in Baf-Sp1 clone 1 (3 independent experiments). Differential gene expression following Sp1 deregulation relative to control was determined using GeneSpring GX 7.3 statistical analysis module ANOVA. The full list of deregulated genes following Sp1 overexpression with informations on each gene (ID, Gene Ontology, Kegg pathway, probeset quality, mean expression, mean deregulation) can be found at ftp://pbil.univ-lyon1.fr/pub/datasets/DeniaudE2009/.

### Endogenous Sp1 is down-regulated immediately following Sp1 overexpression

**Figure 4:**
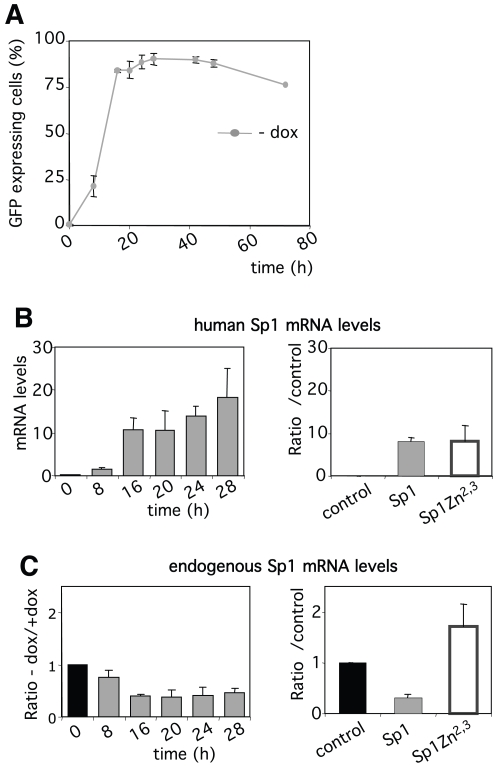
Sp1 autoregulates negatively its transcription. (A) Baf-Sp1 clone 1 was grown in the absence of doxycycline. At the indicated times the percentage of GFP expressing cells was measured by Flow cytometry. (B) & (C) mRNA levels of ectopic human Sp1 and endogenous murine Sp1 mRNA levels measured as indicated in figure 3. Results show the ratio of the mRNA levels measured in Sp1-overexpressing cells relative to mRNA levels measured in control cells and show the mean ± sd of at least 2 independent experiments.

One of the most down-regulated gene following overexpression of human Sp1 is the endogenous murine *sp1* gene (2.3-fold decrease, Table 1). Sp1 promoter contains Sp1 binding sites and the Sp1 protein has been shown to autoregulates its own expression [26]. As Baf-3-Sp1 clone 1 expresses human Sp1 as well as GFP under the same inducible promoter we could monitor, in parallel, GFP protein expression by flow cytometry and both endogenous murine and ectopic human Sp1 mRNA levels by real-time PCR. Kinetic studies showed that the first GFP expressing cells are detected 8 hrs after doxycycline removal (Figure 4A). This correlates with the expression of ectopic Sp1 at the mRNA and protein levels (Figure 4B and data not shown). Importantly, down-regulation of endogenous *sp1* gene transcription starts to be observed as early as 8 hrs after doxycycline removal in the whole cell population where only 20% of the cells are GFP positive (Figure 4C). This down-regulation requires the binding of Sp1 to the DNA. Altogether these results indicate that an excess of DNA-bound Sp1 is sensed by cells and induces a rapid repression of endogenous *sp1* gene expression, revealing a transcriptional negative feedback loop.

### Genes with Sp1 consensus binding site in their promoters are enriched among down-regulated genes but not among up-regulated genes

We next determined whether the set of Sp1-modified genes was enriched for the presence of Sp1 binding motif in their promoters (consensus Sp1 binding motif GGGGCGGGG as defined by TRANFAC 7.0 (V$SP1_Q6) and its variant GG[G/T]G[C/T]GGG [6]). As a control, we also estimated the degree of enrichment for STAT6 transcription factor binding site TTCN_4_GAA [25]. The presence of Sp1 and STAT6 binding sites was searched using a computational approach in the promoter regions consisting of 2000 bp upstream from the transcription start site. As a control, we analysed the promoters of a control list generated randomly that fulfils two criteria: no change in the gene expression levels following Sp1 overexpression and an overall distribution of expression that is similar to the one of the differentially expressed list (Figure S2). There was no significant difference in the percentage of promoters containing STAT6 site among down or up-regulated and control genes. In contrast, Sp1 sites were enriched among the promoters of genes whose expression was repressed (Table 2). Moreover, down-regulated genes contain on average more Sp1 sites in their promoters than genes from the control list. Surprisingly, there was no enrichment for the presence of Sp1 sites among the genes whose expression was increased by Sp1. The *in silico analysis* was extended using comparative genomic tool DiRE that predicts regulatory elements (RE) within the promoters of a set of genes based on their inter-species conservation and their transcription factor binding sites content. Again the percentage of candidate regulatory elements containing a Sp1 binding site was higher within down-regulated genes compared to a control list (Figure S5) and Sp1 was predicted to be the most important transcription factor within the promoters of down-regulated genes. When the analysis was extended to promoters and the most conserved Evolutionary Conserved Regions (ECRs), Sp1 occurrence was the highest among down-regulated genes. Altogether, these analysis indicate Sp1-binding sites are enriched only in down-regulated genes but not in up-regulated genes. This suggests that down-regulation of gene expression could be directly mediated by Sp1 whereas up-regulation of gene expression does not seem to involve the association of Sp1 with proximal promoters. As Sp1 DNA-binding is necessary for deregulation of gene expression, up-regulation of gene expression could represent an indirect event of Sp1 binding to genomic DNA.

**Table 2:** Frequency of Sp1 consensus binding sites in the promoters of genes deregulated by Sp1.

**Sp1 site: GGGGCGGGG**				
	Percentage of genes with binding sites	*p-value* (d)	Number of binding sites per gene	*p-value* (d)
*control list* (a)	15.1%		1.3	
*up-regulated genes* (b)	12.4%	*0.0715*	1.2	*0.2585*
*down-regulated genes* (c)	20.3%	*0.0031*	1.7	*0.0001*
**Variants of Sp1 site: GG[G/T]G[C/T]GGG**				
	Percentage of genes with binding sites	*p-value* (d)	Number of binding sites per gene	*p-value* (d)
*control list* (a)	39.4%		1.7	
*up-regulated genes* (b)	41.3%	*0.3591*	1.7	*0.82*
*down-regulated genes* (c)	47.5%	*0.0005*	2	*0.0004*
**STAT6 site: TTCNNNNGAA**				
	Percentage of genes with binding sites	*p-value* (d)	Number of binding sites per gene	*p-value* (d)
*control list* (a)	41.4%		1.4	
*up-regulated genes* (b)	40.0%	*0.5028*	1.3	*1*
*down-regulated genes* (c)	36.9%	*0.0549*	1.3	*0.1694*

Transcription factor binding sites were searched among the first 2000 nucleotides of the promoter region of differentially expressed genes and non-regulated genes using PATTERNn service. Sp1 binding motifs: GGGGCGGGG and GG[G/T]G[C/T]GGG. STAT6 binding motif: TTCN_4_GAA.

(a) Control list of non-regulated genes generated randomly as described in the experimental procedures.

(b) Up-regulated genes from differentially expressed gene list (>1.3 fold).

(c) Down-regulated genes from differentially expressed gene list (<1.3 fold).

(d) Statistical significance calculated as described in the experimental procedures. P-values are the comparison of up-regulated vs control and down-regulated vs control.

### Deregulated expression of cell cycle regulating genes following Sp1 overexpression is associated with accumulation of cells in the G1 phase of the cell cycle

**Figure 5:**
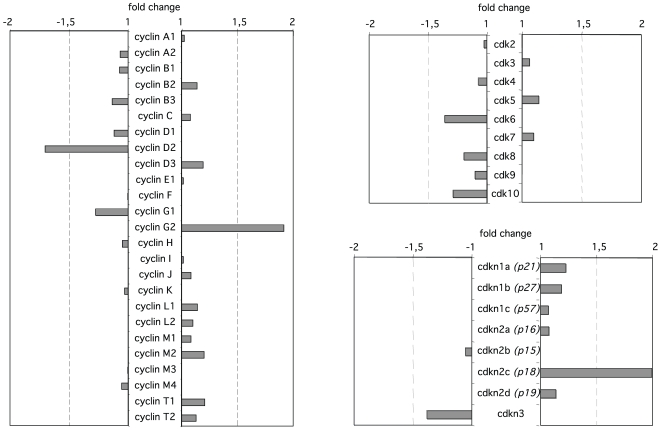
Effect of Sp1 deregulation on the expression of cell cycle regulating genes. Fold change of Cyclins, Cyclin-dependent kinases and Cyclin-dependent kinases inhibitors expression in Sp1 overexpressing cells compared to non expressing cells. Expression ratios are from microarray data (GeneSpring GX).

**Figure 6:**
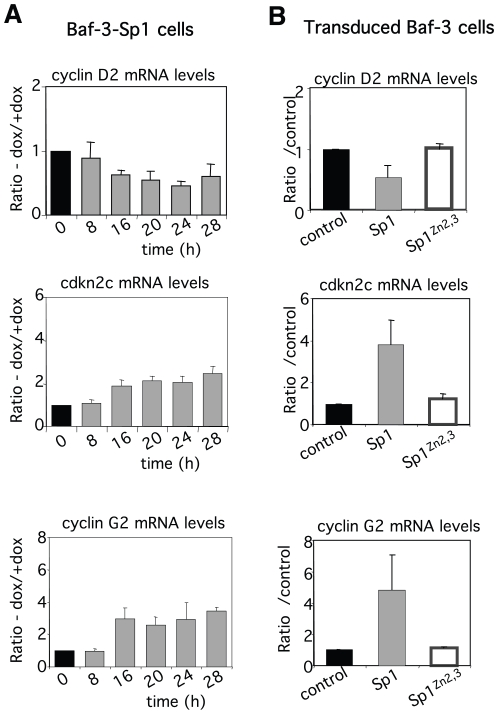
Changes in expression profile of cyclin D2, cdkn2c/p18 and cyclin G2. mRNA levels of cyclin D2, cdkn2c and cyclin G2 in Baf-3-Sp1 clone 1 (A) or in Baf-3 cells transduced with control, Sp1 and Sp1^Zn2,3^ encoding retroviruses (B) were measured by real-time PCR as indicated in figure 3. Results show the ratio of the mRNA levels measured in Sp1-overexpressing cells relative to mRNA levels measured in control cells and show the means of ± sd of at least 2 independent experiments.

**Figure 7:**
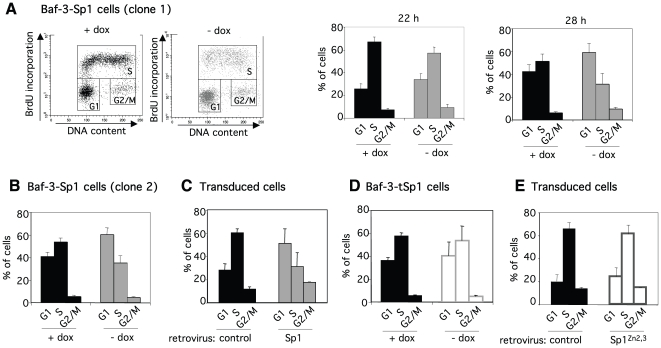
Cell cycle inhibition induced by Sp1 requires its binding to DNA. (A) Baf-3-Sp1 clone 1 was grown in presence of doxycycline (+dox) or in absence of doxycycline for the indicated times (- dox) and pulsed with BrdU for 20 min. Cells were harvested and processed for BrdU labelling and DNA content staining. Cell cycle distribution was analysed by flow cytometry. Representative cell cycle profile with or without doxycycline at 28 hrs (*left panel*). Percentage of cells in the different phases of the cell cycle 22 or 28 hrs after treatment (*right panel*). (B) Cell cycle distribution of Baf-3-Sp1 clone 2 at 72 hrs. (C) Baf-3 cells were transduced with control or Sp1 encoding retrovirus. Cell cycle distribution among transduced (CD2 positive) cells 30 hrs post-infection. (D) Cell cycle distribution of Baf-3-tSp1 clone analysed after 72 hrs. (E) Baf-3 cells transduced with control or Sp1^Zn2,3^ encoding retroviruses were analysed 30 hrs later. Percentage of cells in the different phases of the cell cycle assessed among CD2 positive cells. Results show the means of ± sd of 2 to 3 independent experiments.

Among the genes found to be differentially expressed, using the Ingenuity Pathway Analysis software, we observed the highest enrichments for the following categories: cancer, cell death and cell cycle (Figure S6). The list of genes belonging to these 3 categories being very large, we focused our attention on the cell cycle category as only a very small number of genes involved in the core cell cycle machinery was found to be affected by Sp1 overexpression (Figure 5). As a matter of fact, microarray data indicated that among the genes associated with the progression through the cell cycle such as cyclins, cyclin-dependent protein kinases and cyclin-dependent kinase inhibitors, only three showed a significant modification of expression levels: *Ccnd2* (cyclin D2, 1.7-fold decrease), *Ccng2* (cyclin G2, 1.9-fold increase) and *Cdkn2c* (cyclin-dependent kinase inhibitor 2c/p18, 2.0-fold increase). The down-regulation of *Ccnd2* expression and the up-regulation of *Ccng2* and *Cdkn2c* expression can be observed 16 hrs after doxycycline removal in inducible Baf-3-Sp1 clone (Figure 6A) and require Sp1 binding to DNA (Figure 6B). These genes have all been involved in the regulation of the G1-S transition. Therefore, we next investigated whether Sp1-induced overexpression of these genes could impair cell proliferation. Baf-3-Sp1 clone 1 was grown in the absence or in the presence of doxycycline and the distribution of the cells through the different phases of the cell cycle was studied by flow cytometry after a pulse with BrdU. As soon as 22 hrs after Sp1 induction the percentage of cells in the G1 phase started to increase whereas the percentage of cells in the S phase decreased in comparison to control condition (Figure 7A). A more pronounced effect on cell cycle was observed 28 hrs after Sp1 expression. Similar results were obtained with Baf-3-Sp1 clone 2 (Figure 7B) and in parental Baf-3 cells transduced with wild-type Sp1 (Figure 7C). Cell cycle was not affected by the accumulation of truncated tSp1 in the cytoplasm of the cells (Figure 7D) or by the expression of Sp1^Zn2,3^ (Figure 7E). A detailed analysis of the cell cycle progression indicates that Sp1 overexpression only delays progression from the G1 to the S phase (data not shown). The results indicates that Sp1 overexpression leads to the expression of cell cycle inhibitor Cdkn2c and stress-associated cyclin G2, whereas the expression of cell cycle regulating cyclin D2 is down-regulated and this correlates with the inhibition of G1/S transition of cycling cells, a process that precedes the onset of cell death.

## Discussion

This study extends previous findings that have shown that Sp1 overexpression is detrimental to untransformed cells [18], [19]. Our study shows that overexpressed Sp1 induces an inhibition of the progression through the cell cycle of Baf-3 cells that precedes apoptosis. Both processes require the binding of Sp1 to DNA and are not observed following the expression of a cytoplasmic truncated form of Sp1 or a mutated Sp1 unable to bind DNA. Microarray analysis shows that among the genes found to be differentially expressed following Sp1 overexpression there is an enrichment in genes involved in cancer, cell cycle and cell death.

Indeed, analysis of Sp1-induced deregulated genes with Ingenuity Pathways Analysis software revealed that 223 of them are linked with apoptosis. This includes the up-regulation of proteins that induce apoptosis when overexpressed such as pro-apoptotic Bcl-2 family members (Bmf, Bcl2L11/Bim, Bcl2L13/Rambo) [27], caspase 6 [28], cell death inducing factors (CIDEB, DAP) [29], [30], dependence receptor neogenin [31] (Table 1 and microarray data available in Array express). Therefore Sp1 overexpression could trigger apoptosis through one or a combination of these pathways.

A number of data support a link between Sp1 and cell cycle progression [16], [32]. A computational approach by Elkon et al. comparing the promoters of a set of cell cycle regulated genes revealed that Sp1 binding sequences are significantly enriched in promoters of genes that are expressed in G1/S phase [24]. Although Sp1 has been linked with cell growth, both positive and negative effects of Sp1 on cell growth have been reported. This is consistent with the regulation of both growth promoting genes as well as growth inhibitory genes by Sp1 [16]. Although down-regulation of cell cycle inhibitor cdkn1a/p21 has been implicated in Sp1-induced inhibition of proliferation in vascular smooth muscle cells [19], we did not find a significant modification of *cdkn1a* gene expression upon Sp1 induction in Baf-3 cells (Figure 5). However, we identified *cyclin D2*, *cyclin G2* and *cdkn2c/p18* as being deregulated among cell cycle regulating genes following the binding to DNA of overexpressed Sp1. The products of these genes could be responsible for Sp1-mediated cell cycle inhibition that we observed as they are all involved in regulating G1/S progression. D-cyclins are believed to serve as a link between the extracellular environment and the cell cycle machinery, driving progression through the G1/S phase of the cell cycle. In Baf-3 cells, Interleukin-3 removal induces a decrease of cyclin D2 mRNA expression and a cell cycle arrest that can be abolished by ectopic expression of cyclin D2 [33]. Cdkn2c is a member of the INK4 family that block cell cycle progression by binding to cyclin dependent kinase Cdk4 or Cdk6 and inhibiting the action of cyclin D. Finally, cyclin G2 is an unconventional cyclin that is up-regulated in B cells responding to growth inhibitory signals and its overexpression promotes a cell cycle arrest in G1 phase [34], [35]. These 3 genes have previously been shown to possess Sp1-binding sites in their promoters and/or to be regulated by Sp1 [36]–[38]. In our experimental system, regulation of these cell cycle genes are likely a direct consequence of Sp1 on transcription.

This study revealed that elevation of Sp1 levels is almost immediately followed by a decrease of *sp1* gene transcription. As a matter of fact, endogenous Sp1 mRNA levels start to decrease as soon as 8 hrs after doxycycline removal. At this time point, only 20% of the cells are GFP positive and ectopic Sp1 mRNA starts to be detected in the whole population cell lysate. Transient transfections of Sp1 and luciferase assays using a minimal Sp1 promoter have revealed that Sp1 autoregulates positively its own transcription [26]. Our results confirm that Sp1 autoregulates its own transcription and that an excess of DNA-bound Sp1 represses *sp1* gene transcription in the chromatin context.

The genome-wide expression profiling in Baf-3 cells indicates that a substantial proportion of expressed genes shows a modification of their transcription following Sp1 deregulation. Using real-time PCR, we have confirmed the impact of Sp1 on the expression of 18 out of 18 of these genes and shown that it requires Sp1 binding to DNA (this study and data not shown). To determine the proportion of genes that were potentially transcribed via binding of Sp1 to their promoter, we have search for Sp1-binding sites in the promoter of regulated genes. We observe that only down-regulated genes show a significant enrichment for Sp1 sites in their promoters. It is unlikely that this inhibition is due to the squelching of factors required for transcription as there is no global shut-down of transcription following Sp1 overexpression. Moreover expression of high levels of mutant nuclear form of Sp1 does not lead to transcription modulation. Therefore our results suggest that overexpressed DNA-bound Sp1 exerts a specific inhibitory effect on Sp1-driven transcription in Baf-3 cells. Contrasting with Sp1, the genes induced by c-Rel deregulation show an enrichment for genes containing consensus NF-kB/Rel sites in their proximal promoter region [39]. However, the up-regulation of a large number of genes following Sp1 overexpression could result from an indirect process that requires the binding of Sp1 to DNA. Indeed, DNA-binding of Sp1 could trigger transcription through multiple mechanisms. First, Sp1 could induce the expression of transcription factors that would then activate transcription. This seems unlikely since promoters of up-regulated genes did not exhibit an enrichment for binding sites for the few transcription factors (c-jun, Fos, E2F2) that were up-regulated by Sp1 (data not shown). Moreover, the DiRE analysis does not reveal an enrichment of putative regulatory elements in the non-coding sequence of deregulated genes (Figure S5). Second, Sp1 might affect gene transcription through the control of non coding RNA as it was observed that many Sp1 binding sites are localised next to non coding RNA genes [6]. Finally, the human genome contains more than 12,000 occupied Sp1 binding sites [6] and Sp1 has also been shown to localize within subnuclear foci that infrequently overlap with sites of transcription [40]. Therefore, the association of deregulated Sp1 with DNA might perturb chromatin structure. Further experiments will be necessary to determine the precise mechanisms involved in the gene transcription up-regulation induced by overexpressed Sp1.

Deregulated expression of Sp1 has been associated with the late steps of cell transformation [14]–[16]. Analysis of the categories of genes enriched following Sp1 deregulation revealed that the highest enrichment was observed for genes belonging to the Cancer category (Figure S6). The expression of genes known to have important role in migration/metastasis were up-regulated following Sp1 overexpression. This includes actin-binding protein gelsolin a protein involved in cell motility and required for cancer cell invasion [41], matrix metalloproteases MMP9 which degrade extracellular matrix and promotes invasion [42], metastasis-associated proteins S100A4/metastasin [43] and cathepsin B [44] (data not shown). Altogether these results show that Sp1 overexpression is detrimental for untransformed cells. However, in cells having acquired resistance to cell cycle inhibition and cell death, expression of these genes involved in migration/metastasis could confer a selective advantage and contribute to their further transformation.

## Supporting Information

Figure S1 Sp1 constructs used in the study. Wild-type of Sp1 (Sp1) consists of four transactivation domains (A to D) and a DNA-binding domain (DBD) composed of three Zinc fingers. The mutant form Sp1Zn2,3 carries point mutation on the second and third Zinc fingers of the DBD. The truncated Sp1 tSp1 is composed of the first 418 amino acids.
(0.28 MB EPS)

Figure S2 Distribution of the expression of the genes differentially expressed following Sp1 deregulation (1610 probesets) (A) and the non-regulated genes from a control list (1700 probesets) (B) generated as indicated in the experimental procedures.
(0.38 MB EPS)

Figure S3 Zn finger mutations abolish Sp1 ability to induce apoptosis. Baf-3 cells were transduced with retroviruses encoding wild-type Sp1 (Sp1) or Sp1 mutants carrying mutations in their DNA binding domain and analysed 30 hrs later. (A) Cells were costained for actin (red), Sp1 (green) and DNA (blue) and analysed by confocal microscopy. Arrowheads indicate overexpressing cells. Scale bar, 10 µm. (B) Left Western blot analysis of Sp1 protein levels in Baf-3 cells transduced with either control, Sp1 or various Sp1Zn mutants retroviruses purified with anti-CD2 antibody 30 hrs post-infection. Right The percentage of apoptotic cells (Annexin V positive) was measured at various time points among infected cells (CD2 positive) by flow cytometry. Results show the mean ± sd of at least 2 independent experiments.
(0.77 MB EPS)

Figure S4 Gene expression profiles following Sp1 overexpression. Scatter plots of the mean signal intensity values of three independent microarray experiments (Affymetrix Mouse Genome 430.2). Data were analysed using GeneSpring GX 7.3 expression analysis software (Agilent Technology Inc). (A) All probesets. (B) Probesets differentially expressed following Sp1 overexpression relative to control. Differential expression was determined using GeneSpring GX 7.3 statistical analysis module ANOVA 1 way test, assuming variance not equal, choosing a false discovery rate of 6% with multiple testing correction (Benjamini and Hochberg). This gave a list of 1610 differentially expressed probesets with a fold change of expression of 1.3.
(0.44 MB EPS)

Figure S5 Analysis of transcription factor binding sites within Regulatory Elements (RE), predicted using inter-species conservation, of (A) down-regulaled genes, (B) up-regulated genes and (C) the control list (expressed but non- regulated). Search was performed via the DiRE server which uses RE located within as well outside promoters (http://dire.dcode.org, Gotea, V. and I. Ovcharenko (2008) DiRE: identifying distant regulatory elements of co-expressed genes. Nucleic Acids Research, doi:10.1093/nar/gkn300). The results show the occurence of transcription factor binding sites within RE and the Importance of each transcription factor that is the product of its occurrence and its weight (i.e. its association with the input gene set). The number of genes eligible for DiRE analysis are indicated.
(0.36 MB EPS)

Figure S6 Functional classification of differentially expressed genes following Sp1 overexpression. Functional classification was performed using the Ingenuity Pathway Analysis software. Among the genes found to be differentially expressed with a fold change of expression of 1.3, 732 genes were eligible for function/pathway analysis. The seven highest-ranking functional categories are shown. The scores are based on their connectivity.
(0.34 MB EPS)
